# Performance Analysis of Several GPS/Galileo Precise Point Positioning Models

**DOI:** 10.3390/s150614701

**Published:** 2015-06-19

**Authors:** Akram Afifi, Ahmed El-Rabbany

**Affiliations:** Department of Civil Engineering, Ryerson University, Toronto, ON M5B 2K3, Canada; E-Mail: rabbany@ryerson.ca

**Keywords:** ambiguity, PPP, GPS, Galileo, BSSD, decoupled clock

## Abstract

This paper examines the performance of several precise point positioning (PPP) models, which combine dual-frequency GPS/Galileo observations in the un-differenced and between-satellite single-difference (BSSD) modes. These include the traditional un-differenced model, the decoupled clock model, the semi-decoupled clock model, and the between-satellite single-difference model. We take advantage of the IGS-MGEX network products to correct for the satellite differential code biases and the orbital and satellite clock errors. Natural Resources Canada’s GPSPace PPP software is modified to handle the various GPS/Galileo PPP models. A total of six data sets of GPS and Galileo observations at six IGS stations are processed to examine the performance of the various PPP models. It is shown that the traditional un-differenced GPS/Galileo PPP model, the GPS decoupled clock model, and the semi-decoupled clock GPS/Galileo PPP model improve the convergence time by about 25% in comparison with the un-differenced GPS-only model. In addition, the semi-decoupled GPS/Galileo PPP model improves the solution precision by about 25% compared to the traditional un-differenced GPS/Galileo PPP model. Moreover, the BSSD GPS/Galileo PPP model improves the solution convergence time by about 50%, in comparison with the un-differenced GPS PPP model, regardless of the type of BSSD combination used. As well, the BSSD model improves the precision of the estimated parameters by about 50% and 25% when the loose and the tight combinations are used, respectively, in comparison with the un-differenced GPS-only model. Comparable results are obtained through the tight combination when either a GPS or a Galileo satellite is selected as a reference.

## 1. Introduction

**Figure 1:**
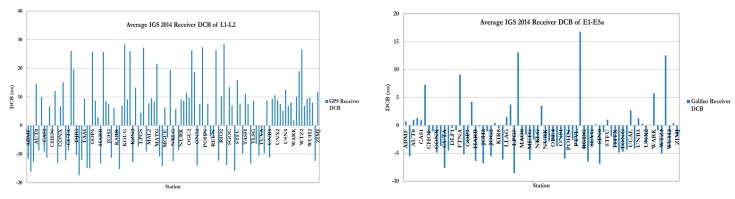
Average 2014 IGS receiver DCB for GPS and Galileo signals.

**Figure 2:**
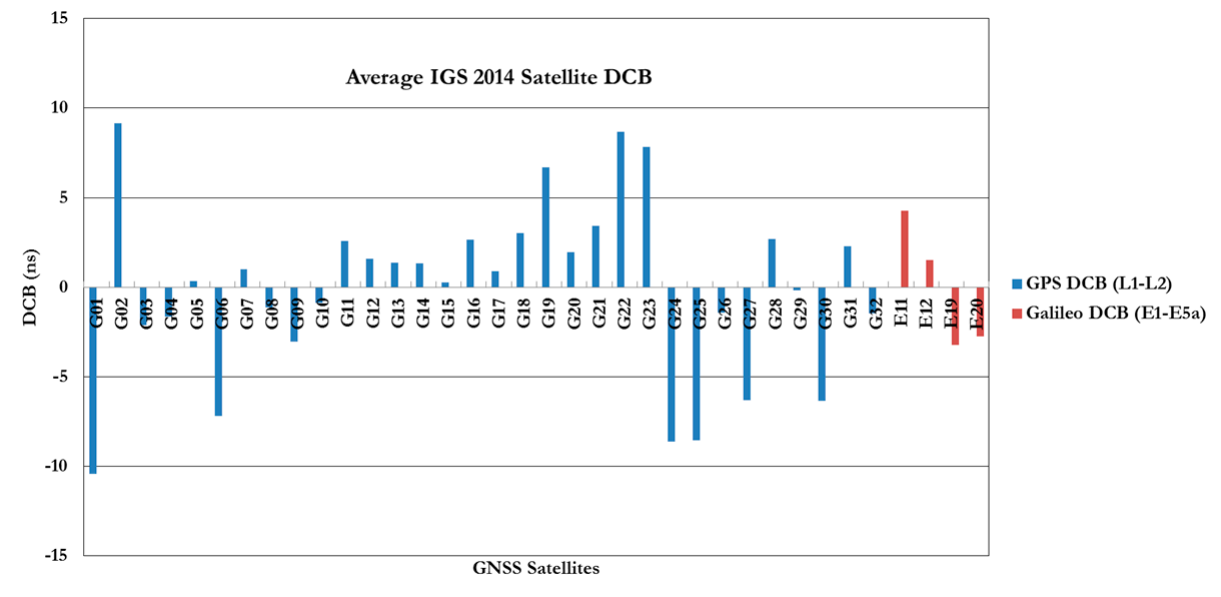
Average IGS 2014 satellite DCB for both GPS/Galileo signals.

GNSS precise point positioning (PPP) has proven to be capable of providing positioning accuracy at the sub-decimeter and decimeter levels in static and kinematic modes, respectively. PPP accuracy and convergence time are controlled by the ability to mitigate all potential error biases in the system. Several comprehensive studies have been published on the accuracy and convergence time of un-differenced GPS and GPS/Galileo PPP models (see for example, [1,2,3,4,5,6]. In the traditional un-differenced GPS PPP model, because of the presence of the un-calibrated hardware delays, the ambiguity parameters are typically obtained as real-value numbers [4,5,7,8]. This in turn affects the GPS PPP solution convergence and accuracy [9]. However, recent research has demonstrated that the correct integer values for the ambiguity parameters can be recovered if the satellite hardware delays can be calibrated. Figure 1 and Figure 2 show the IGS average estimated values of the receiver and satellite differential code biases, respectively, for 2014 [10]. As can be seen in Figure 2, Galileo satellite differential code biases of E1/E5a signals are relatively smaller than the GPS L1/L2 counterpart.

For a single GNSS constellation, between-satellite single-difference (BSSD) linear combination cancels out all receiver-related errors, including the receiver hardware delays, which significantly improves the convergence time [3,4,11,12]. This, however, is not the case when the measurements of two or more constellations are combined. When forming BSSD for GPS and Galileo measurements, three scenarios can be considered on the selection of the reference satellite. Either a GPS or a Galileo satellite is selected as a reference for both GPS and Galileo observables. Alternatively, two reference satellites are selected: a GPS reference satellite for the GPS observables and a Galileo satellite for the Galileo observables. The first approach is commonly referred to as tight combination, while the latter is commonly referred to as per-constellation or loose combination [6].

This paper examines the performance of several PPP models, which combine the dual-frequency GPS/Galileo observables in both un-differenced and BSSD mode. The IGS-MGEX network products used to correct for the satellite differential code biases, the orbital and satellite clock errors [13]. As the IGS-MGEX products are presently referenced to the GPS time and since we use mixed GNSS receivers that also use the GPS time as a reference, the GPS to Galileo time offset (GGTO) is cancelled out in our models. The inter-system bias is either cancelled out through differencing the observations or is treated as an additional unknown parameter. The Hopfield tropospheric correction model is used, along with the Vienna mapping function, to account for the hydrostatic component of the tropospheric delay [14,15]. The wet component is treated as an additional unknown parameter in the estimation model. Other corrections are also applied, including the effect of ocean loading [16,17], Earth tide [18], carrier-phase windup [19,20], Sagnac [21], relativity [22], and satellite and receiver antenna phase-center variations [23]. Natural Resources Canada’s GPSPace PPP software is modified to handle the various GPS/Galileo PPP models. A total of six data sets of GPS and Galileo observations at six IGS stations are processed to examine the performance of the various PPP models. It is shown that the traditional un-differenced GPS/Galileo PPP model, the GPS decoupled clock model, and semi-decoupled clock GPS/Galileo PPP model improve the convergence time by about 25% in comparison with the un-differenced GPS-only model. In addition, the semi-decoupled GPS/Galileo PPP model improves the solution precision by about 25% compared to the traditional un-differenced GPS/Galileo PPP model. Moreover, the BSSD GPS/Galileo PPP model improves the solution convergence time by about 50%, in comparison with the un-differenced GPS PPP model, regardless of the type of BSSD combination used. As well, the BSSD model improves the precision of the estimated parameters by about 50% and 25% when the loose and the tight combinations are used, respectively, in comparison with the un-differenced GPS-only model. Comparable results are obtained through the tight combination when either a GPS or a Galileo satellite is selected as a reference.

## 2. Un-Differenced GPS/Galileo PPP Models

### 2.1. Traditional GPS/Galileo PPP Model

PPP has traditionally been carried out using dual-frequency ionosphere-free linear combinations of carrier-phase and pseudorange GPS measurements. Equations (1)–(4) show the ionosphere free linear combination of both GPS/Galileo observations [6]:

(1) $$P_{G_{IF}}=\rho_{G}+c\text{[}dt_{rG}-dt^{s}\text{]}+c{\text{[}\alpha d_{P1}-\beta d_{P2}\text{]}}_{r}+c{\text{[}\alpha d_{P1}-\beta d_{P2}\text{]}}^{s}+T_{G}+\varepsilon_{PG_{IF}}$$

(2) $$P_{E_{IF}}=\rho_{E}+c[dt_{rG}-GGTO-dt^{s}]+c{\left[\alpha d_{E1}-\beta d_{E5a}\right]}_{r}+c{\left[\alpha d_{E1}-\beta d_{E5a2}\right]}^{s}+T_{E}+\varepsilon_{E_{IF}}$$

(3) $$\Phi_{G_{IF}}=\rho_{G}+c[dt_{rG}-dt^{s}]+c{\left[\alpha \delta_{L1}-\beta \delta_{L2}\right]}_{r}+c{\left[\alpha \delta_{L1}-\beta \delta_{L2}\right]}^{s}+T_{G}+N_{G_{IF}}+\phi_{r0_{G_{IF}}}+\phi_{0_{G_{IF}}}^{s}+\varepsilon_{\Phi G_{IF}}$$

(4) $$\Phi_{E_{IF}}=\rho_{E}+c[dt_{rG}-GGTO-dt^{s}]+c{\left[\alpha \delta_{E1}-\beta \delta_{E5a}\right]}_{r}+c{\left[\alpha \delta_{E1}-\beta \delta_{E5a}\right]}^{s}+T_{E}+N_{E_{IF}}+\phi_{r0_{E_{IF}}}+\phi_{0_{E_{IF}}}^{s}+\varepsilon_{\Phi E_{IF}}$$

where the subscripts G and E refer to the GPS and Galileo satellite systems, respectively; $P_{G_{IF}}$ and $P_{E_{IF}}$ are the ionosphere-free pseudoranges in meters for GPS and Galileo systems, respectively; $\Phi_{G_{IF}}$ and $\Phi_{E_{IF}}$ are the ionosphere-free carrier phase measurements in meters for GPS and Galileo systems, respectively; ρ is the true geometric range from receiver at reception time to satellite at transmission time in meter; *dt_r_*, *dt^s^* are the clock errors in seconds for the receiver at signal reception time and the satellite at signal transmission time, respectively; $d_{P1r}$, $d_{P2r}$, $d_{E1r}$, $d_{E5ar}$ are frequency-dependent code hardware delays for the receiver at reception time in seconds; ${d_{P1}}^{S}$, ${d_{P2}}^{S}$, ${d_{E1}}^{S}$, ${d_{E5a}}^{S}$ are frequency-dependent code hardware delays for the satellite at transmission time in seconds; $\delta_{L1}$, $\delta_{L2}$, $\delta_{E1}$, $\delta_{E5a}$ are frequency-dependent carrier-phase hardware delays for the receiver at reception time in seconds; ${\delta_{L1}}^{S}$, ${\delta_{L2}}^{S}$, ${\delta_{E1}}^{S}$, ${\delta_{E5a}}^{S}$ are frequency-dependent carrier-phase hardware delays for the satellite at transmission time in seconds; *T* is the tropospheric delay in meter; $N_{G_{IF}}$, $N_{E_{IF}}$ are the ionosphere-free linear combinations of the ambiguity parameters for both GPS and Galileo carrier-phase measurements in meters, respectively (Equations (5) and (6)); $\phi_{r0_{G_{IF}}}$, $\phi_{0_{G_{IF}}}^{S}$, $\phi_{r0_{E_{IF}}}$, $\phi_{0_{E_{IF}}}^{S}$ are ionosphere-free linear combinations of frequency-dependent initial fractional phase biases in the receiver and satellite channels for both GPS and Galileo in meters, respectively; *c* is the speed of light in vacuum in meter per second; $\varepsilon_{P_{IF}}$, $\varepsilon_{E_{IF}}$, $\varepsilon_{\Phi G_{IF}}$, $\varepsilon_{\Phi E_{IF}}$ are the ionosphere-free linear combinations of the relevant noise and un-modeled errors in meter; $\alpha_{G}$, $\beta_{G}$, $\alpha_{E}$, $\beta_{E}$ are the ionosphere-free linear combination coefficients for both GPS and Galileo, which are given, respectively, by: $\alpha_{G}=\frac{f_{1}^{2}}{f_{1}^{2}-f_{2}^{2}}$, $\beta_{G}=\frac{f_{2}^{2}}{f_{1}^{2}-f_{2}^{2}}$, $\alpha_{E}=\frac{f_{E1}^{2}}{f_{E1}^{2}-f_{E5a}^{2}}$, $\beta_{E}=\frac{f_{E5a}^{2}}{f_{E1}^{2}-f_{E5a}^{2}}$, where *f_1_* and *f_2_* are GPS L_1_ and L_2_ signals frequencies; *f_E1_* and *f_E5a_* are Galileo E_1_ and E_5a_ signals frequencies:

(5) $$N_{G_{IF}}=\alpha_{G}\lambda_{1}N_{1}-\beta_{G}\lambda_{2}N_{2}$$

(6) $$N_{E_{IF}}=\alpha_{E}\lambda_{E1}N_{E1}-\beta_{E}\lambda_{E5a}N_{E5a}$$

where λ*_1_* and λ*_2_* are wavelengths of the GPS L1 and L2 signals, respectively, in meters; λ*_E1_* and λ*_E5a_* are the Galileo E1 and E5a signals wavelengths in meters; *N_1_*, *N_2_* are the integer ambiguity parameters of GPS signals L1 and L2, respectively; *N_E1_*, *N_E5a_* are the integer ambiguity parameters of Galileo signals E1 and E5a, respectively.

As indicated earlier, precise orbit and satellite clock corrections from the IGS-MGEX network are used to correct both of the GPS and Galileo measurements. It should be pointed out that such products are presently referenced to the GPS time frame [24]. As well, the IGS-MGEX precise GPS satellite clock corrections include the effect of the ionosphere-free linear combination of the satellite hardware delays of L1/L2 *P*(Y) code, while the Galileo counterpart include the effect of the ionosphere-free linear combination of the satellite hardware delays of the Galileo E1/E5a pilot code [24]. Applying the precise clock corrections to Equations (1)–(4), we obtain:

(7) $$P_{G_{IF}}=\rho_{G}+c[dt_{rG}-dt_{prec}^{s}]+c{\left[\alpha d_{P1}-\beta d_{P2}\right]}_{r}+T_{G}+\varepsilon_{PG_{IF}}$$

(8) $$P_{E_{IF}}=\rho_{E}+c[dt_{rG}-dt_{prec}^{s}]+c{\left[\alpha d_{E1}-\beta d_{E5a}\right]}_{r}+T_{E}+\varepsilon_{E_{IF}}$$

(9) $$\Phi_{G_{IF}}=\rho_{G}+cdt_{rG}-c[dt_{prec}^{s}+{\left[\alpha d_{P1}-\beta d_{P2}\right]}^{s}]+c{\left[\alpha \delta_{L1}-\beta \delta_{L2}\right]}_{r}-c{\left[\alpha \delta_{L1}-\beta \delta_{L2}\right]}^{s}+T_{G}+N_{G_{IF}}+\phi_{r0_{G_{IF}}}+\phi_{0_{G_{IF}}}^{s}+\varepsilon_{\Phi G_{IF}}$$

(10) $$\Phi_{E_{IF}}=\rho_{E}+cdt_{rG}-c\text{[}dt_{prec}^{s}+{\text{[}\alpha d_{E1}-\beta d_{E5a}\text{]}}^{s}\text{]}+c{\text{[}\alpha \delta_{E1}-\beta \delta_{E5a}\text{]}}_{r}-c{\text{[}\alpha \delta_{E1}-\beta \delta_{E5a}\text{]}}^{s}+T_{E}+N_{E_{IF}}+\phi_{r0_{E_{IF}}}+\phi_{0_{E_{IF}}}^{s}+\varepsilon_{\Phi E_{IF}}$$

For simplicity, the receiver and satellite hardware delays will take the following forms: $b_{r_{P}}=c{\left[\alpha d_{P1}-\beta d_{P2}\right]}_{r}$$b_{P}^{s}=c{\left[\alpha d_{P1}-\beta d_{P2}\right]}^{s}$$b_{r_{E}}=c{\left[\alpha d_{E1}-\beta d_{E5a}\right]}_{r}$$b_{E}^{s}=c{\left[\alpha d_{E1}-\beta d_{E5a}\right]}^{s}$$b_{r_{\Phi }}=c{\left[\alpha \delta_{L1}-\beta \delta_{L2}\right]}_{r}+\phi_{r0_{G_{IF}}}$$b_{\Phi }^{s}=c{\left[\alpha \delta_{L1}-\beta \delta_{L2}\right]}^{s}+\phi_{0_{G_{IF}}}^{s}$$b_{r_{E\Phi }}=c{\left[\alpha \delta_{E1}-\beta \delta_{E5a}\right]}_{r}+\phi_{r0_{E_{IF}}}$$b_{E\Phi }^{s}=c{\left[\alpha \delta_{E1}-\beta \delta_{E5a}\right]}^{s}+\phi_{0_{E_{IF}}}^{s}$

In the traditional GPS/Galileo un-differenced PPP model, the GPS receiver clock error is lumped to the GPS receiver differential code biases. In order to maintain consistency in the estimation of a common receiver clock offset, this convention is used when combining the ionosphere-free linear combination of GPS L1/L2 and Galileo E1/E5a observations. This, however, introduces an additional bias in the Galileo ionosphere-free PPP mathematical model, which represents the difference in the receiver differential code biases of both systems. Such an additional bias is commonly known as the inter-system bias (ISB). With the above consideration, the GPS/Galileo ionosphere-free linear combinations of the pseudorange and carrier-phase observations can be written as:

(11) $$P_{G_{IF}}=\rho_{G}+\tilde{d}t_{rG}-dt_{prec}^{s}+T_{G}+\varepsilon_{PG_{IF}}$$

(12) $$P_{E_{IF}}=\rho_{E}+\tilde{d}t_{rG}-dt_{prec}^{s}+ISB+T_{E}+\varepsilon_{E_{IF}}$$

(13) $$\Phi_{G_{IF}}=\rho_{G}+\tilde{d}t_{rG}-dt_{prec}^{s}+T_{G}+{\tilde{N}}_{G_{IF}}+\varepsilon_{\Phi G_{IF}}$$

(14) $$\Phi_{E_{IF}}=\rho_{E}+\tilde{d}t_{rG}-dt_{prec}^{s}+T_{E}+{\tilde{N}}_{E_{IF}}+ISB+\varepsilon_{\Phi E_{IF}}$$

where $\tilde{d}t_{rG}$ represents the sum of the receiver clock error and receiver hardware delay $\tilde{d}t_{rG}=cdt_{rG}+b_{r_{P}}$; $dt_{prec}^{s}$ is the precise satellite clock correction; *ISB* is the inter-system bias, $ISB=b_{r_{E}}-b_{r_{P}}$; ${\tilde{N}}_{G_{IF}}$ and ${\tilde{N}}_{E_{IF}}$ are given by:

(15) $${\tilde{N}}_{G_{IF}}=N_{G_{IF}}+b_{r_{\Phi }}+b_{r_{P}}-b_{\Phi }^{s}-b_{P}^{s}$$

(16) $${\tilde{N}}_{E_{IF}}=N_{E_{IF}}+b_{r_{E\Phi }}+b_{r_{P}}-b_{E\Phi }^{s}-b_{E}^{s}$$

When using the traditional un-differenced GPS/Galileo PPP model, the ambiguity parameters lose its integer nature as they are contaminated by the receiver and satellite hardware delays.

### 2.2. Decoupled Clock GPS/Galileo PPP Model

The decoupled clock model assigns two different receiver and satellite clocks for the pseudorange and carrier-phase measurements [25]. Applying the concept of the decoupled clock on the combined GPS and Galileo measurements and using Equations (1)–(4), we obtain:

(17) $$P_{G_{IF}}=\rho_{G}+c[dt_{rG}-dt_{G}^{S}]+c{\left[\alpha d_{P1}-\beta d_{P2}\right]}_{r}+c{\left[\alpha d_{P1}-\beta d_{P2}\right]}^{s}+T_{G}+\varepsilon_{PG_{IF}}$$

(18) $$P_{E_{IF}}=\rho_{E}+c[dt_{rE}-GGTO-dt_{E}^{S}]+c{\left[\alpha d_{E1}-\beta d_{E5a}\right]}_{r}+c{\left[\alpha d_{E1}-\beta d_{E5a2}\right]}^{s}+T_{E}+\varepsilon_{E_{IF}}$$

(19) $$\Phi_{G_{IF}}=\rho_{G}+c\text{[}dt_{rG\Phi }-dt_{G\Phi }^{S}\text{]}+c{\text{[}\alpha \delta_{L1}-\beta \delta_{L2}\text{]}}_{r}+c{\text{[}\alpha \delta_{L1}-\beta \delta_{L2}\text{]}}^{s}+T_{G}+N_{G_{IF}}+\phi_{r0_{G_{IF}}}+\phi_{0_{G_{IF}}}^{s}+\varepsilon_{\Phi G_{IF}}$$

(20) $$\Phi_{E_{IF}}=\rho_{E}+c\text{[}dt_{rE\Phi }-GGTO-dt_{E\Phi }^{S}\text{]}+c{[\alpha \delta_{E1}-\beta \delta_{E5a}\text{]}}_{r}+c{\text{[}\alpha \delta_{E1}-\beta \delta_{E5a}\text{]}}^{s}+T_{E}+N_{E_{IF}}+\phi_{r0_{E_{IF}}}+\phi_{0_{E_{IF}}}^{s}+\varepsilon_{\Phi E_{IF}}$$

To simplify Equations (17)–(20), the receiver and satellite clock errors can be written as: $d{\tilde{t}}_{r_{G}}=cdt_{r_{G}}+b_{r_{P}}$$d{\tilde{t}}_{G}^{s}=cdt_{G}^{s}+b_{P}^{S}$$d{\tilde{t}}_{rE}=cdt_{rE}+b_{r_{E}}$$d{\tilde{t}}_{E}^{s}=cdt_{E}^{s}+b_{E}^{S}$$d{\tilde{t}}_{rG\Phi }=cdt_{rG\Phi }+b_{r\Phi }$$d{\tilde{t}}_{G\Phi }^{s}=cdt_{G\Phi }^{s}+b_{\Phi }^{S}$$d{\tilde{t}}_{rE\Phi }=cdt_{rE\Phi }+b_{r_{E\Phi }}$$d{\tilde{t}}_{E\Phi }^{s}=cdt_{\Phi }^{s}+b_{E\Phi }^{S}$ where $d{\tilde{t}}_{G}^{s}$, $d{\tilde{t}}_{E}^{s}$, $d{\tilde{t}}_{G\Phi }^{s}$, and $d{\tilde{t}}_{E\Phi }^{s}$ are the decoupled satellite clock errors for the pseudorange and carrier phase measurements of both GPS and Galileo systems, respectively. $d{\tilde{t}}_{r_{G}}$, $d{\tilde{t}}_{r_{E}}$, $d{\tilde{t}}_{rG\Phi }$, and $d{\tilde{t}}_{rE\Phi }$ are the receiver clock errors for the pseudorange and carrier phase measurements of both GPS and Galileo systems, respectively

In the decoupled clock PPP model, the initial phase bias is lumped to the receiver hardware delays. As such, Equations (17)–(20) can be re-written as follows:

(21) $$P_{G_{IF}}=\rho_{G}+d{\tilde{t}}_{rG}-d{\tilde{t}}_{G}^{S}+T_{G}+\varepsilon_{PG_{IF}}$$

(22) $$P_{E_{IF}}=\rho_{E}+d{\tilde{t}}_{rE}-d{\tilde{t}}_{E}^{S}+T_{E}+\varepsilon_{E_{IF}}$$

(23) $$\Phi_{G_{IF}}=\rho_{G}+d{\tilde{t}}_{rG\Phi }-d{\tilde{t}}_{G\Phi }^{S}+T_{G}+N_{G_{IF}}+\varepsilon_{\Phi G_{IF}}$$

(24) $$\Phi_{E_{IF}}=\rho_{E}+d{\tilde{t}}_{rE\Phi }-d{\tilde{t}}_{E\Phi }^{S}+T_{E}+N_{E_{IF}}+\varepsilon_{\Phi E_{IF}}$$

As shown in Equations (21)–(24), the assumption of having a separate receiver clock error for the pseudorange and the carrier phase observables is more complex in the case of GPS/Galileo PPP model. As all the observables are collected through a single receiver, which uses one time scale, it is uncommon to have a receiver clock error for each constellation and for each observable. As such, only the GPS receiver clock error for both of the pseudorange and carrier phase measurements is considered, assuming that the receiver uses the GPS time as a reference. Therefore, an inter-system bias term appears in the Galileo pseudorange and carrier phase equations to represent the difference between the GPS and Galileo receiver hardware delays. This leads to:

(25) $$P_{G_{IF}}=\rho_{G}+d{\tilde{t}}_{rG}-d{\tilde{t}}_{G}^{S}+T_{G}+\varepsilon_{PG_{IF}}$$

(26) $$P_{E_{IF}}=\rho_{E}+d{\tilde{t}}_{rG}-d{\tilde{t}}_{E}^{S}+ISB_{P}+T_{E}+\varepsilon_{E_{IF}}$$

(27) $$\Phi_{G_{IF}}=\rho_{G}+d{\tilde{t}}_{rG\Phi }-d{\tilde{t}}_{G\Phi }^{S}+T_{G}+N_{G_{IF}}+\varepsilon_{\Phi G_{IF}}$$

(28) $$\Phi_{E_{IF}}=\rho_{E}+d{\tilde{t}}_{rG\Phi }-d{\tilde{t}}_{E\Phi }^{S}+ISB_{C}+T_{E}+N_{E_{IF}}+\varepsilon_{\Phi E_{IF}}$$

where the *ISB_P_* and *ISB_C_* are the pseudorange and carrier-phase inter-system biases, respectively, which are given by: $ISB_{P}=b_{r_{E\Phi }}-b_{r_{\Phi }}$ and $ISB_{C}=b_{r_{E}}-b_{r_{P}}$.

Equations (25)–(28) can be re-arranged by considering the satellite clock corrections and the dry tropospheric correction as follows:

(29) $$\rho_{G}+d{\tilde{t}}_{rG}+m_{f}zpd_{w}+\varepsilon_{PG_{IF}}-{\tilde{P}}_{G_{IF_{DC}}}=0$$

(30) $$\rho_{E}+d{\tilde{t}}_{rG}+m_{f}zpd_{w}+ISB_{P}+\varepsilon_{E_{IF}}-{\tilde{P}}_{E_{IF_{DC}}}=0$$

(31) $$\rho_{G}+d{\tilde{t}}_{rG\Phi }+m_{f}zpd_{w}+N_{G_{IF}}+\varepsilon_{\Phi G_{IF}}-\Phi_{G_{IF_{DC}}}=0$$

(32) $$\rho_{E}+d{\tilde{t}}_{rG\Phi }+m_{f}zpd_{w}+N_{E_{IF}}+ISB_{C}+\varepsilon_{\Phi E_{IF}}+\Phi_{E_{IF_{DC}}}=0$$

where, ${\tilde{P}}_{G_{IF_{DC}}}$, ${\tilde{P}}_{E_{IF_{DC}}}$, ${\tilde{\Phi }}_{G_{IF_{DC}}}$ and ${\tilde{\Phi }}_{E_{IF_{DC}}}$ are the ionosphere-free linear combinations of the pseudorange and carrier-phase observables after applying the above corrections; *zpd_w_* is the wet component of the tropospheric zenith path delay; *m_f_* troposphere mapping functions; The ambiguity parameters of the decoupled clock PPP model are given by:

(33) $$N_{G_{IF}}=\lambda_{G_{IF}}[f_{1}N_{1}-f_{2}N_{2}]$$

(34) $$N_{E_{IF}}=\lambda_{E_{IF}}[f_{E1}N_{1}-f_{E5a}N_{2}]$$

**Figure 3:**
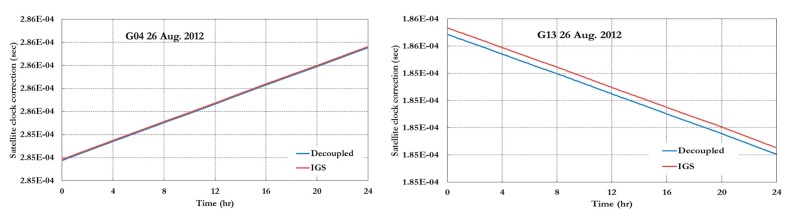

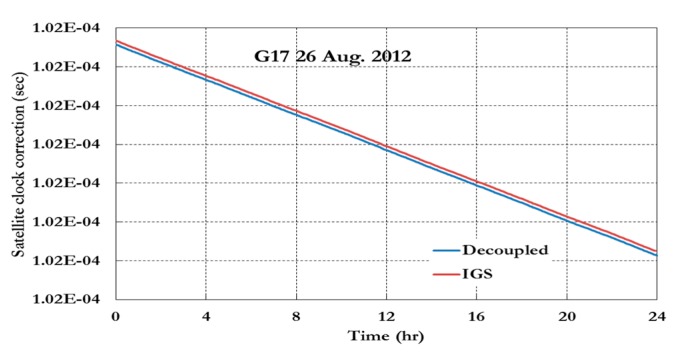
IGS and decoupled clock corrections 26 August 2012.

**Figure 4:**
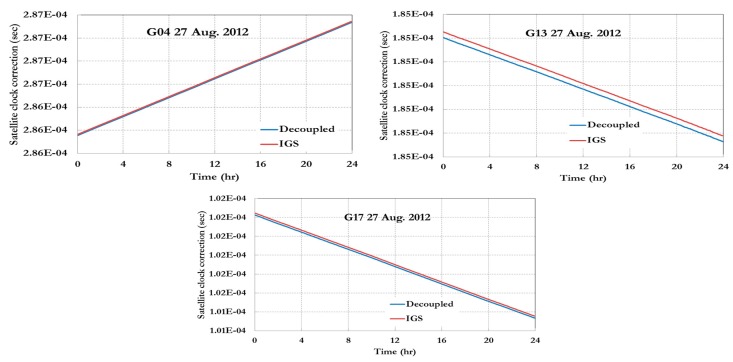
IGS and decoupled clock corrections 27 August 2012.

**Figure 5:**
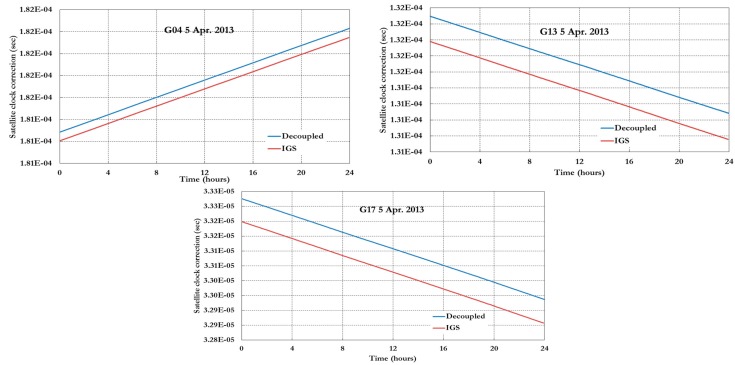
IGS and decoupled clock corrections 5 April 2013.

Figure 3, Figure 4 and Figure 5 show the decoupled precise satellite clock corrections for the pseudorange and carrier-phase observations for different days, namely 26–27 August 2012, and 5 April 2013. As indicated earlier, the difference between the decoupled satellite clock corrections is the satellite hardware delay for pseudorange and carrier phase observations as shown in Figure 3, Figure 4 and Figure 5. Only the GPS decoupled clock products are presented in this paper because of the unavailability of the Galileo decoupled clock products at present.

As shown in Figure 3 and Figure 4, the difference between the IGS (pseudorange) and decoupled (carrier phase) clock corrections is essentially constant. However, in Figure 5, which the data used represent around 7 months after the data used for Figure 3 and Figure 4, the difference between the IGS and decoupled clock corrections is different than the ones in Figure 3 and Figure 4 as shown in Table 1.

As shown in Table 1, the difference between the IGS and decoupled precise clock correction can be assumed to be constant for the short period of time extent to days; however, for long term cases the difference will not be constant. As a result, it can be concluded that either satellite clock corrections or un-calibrated satellite hardware delays drift over time or even both change over time.

**Table 1:** Satellite clock correction difference between decoupled and IGS products.

Date	Satellite Clock Correction Difference (Decoupled-IGS) (s)		
	G04	G13	G17
**26 August 2012**	−6.91E−09	−1.20E−08	−5.60E−09
**27 August 2012**	−6.91E−09	−1.20E−08	−5.60E−09
**5 April 2013**	8.19E−08	7.99E−08	7.83E−08

### 2.3. Semi-Decoupled Clock GPS/Galileo PPP Model

In this model, the IGS-MGEX network precise clock corrections and the daily DCB for both GPS/Galileo satellites are used [10,13]. Considering the carrier-phase DCB, Equations (29)–(31) can be rewritten as:

(35) $$\rho_{G}+d{\tilde{t}}_{rG}+m_{f}zpd_{w}+\varepsilon_{PG_{IF}}-{\tilde{P}}_{G_{IF}}=0$$

(36) $$\rho_{E}+d{\tilde{t}}_{rG}+m_{f}zpd_{w}+ISB_{P}+\varepsilon_{E_{IF}}-{\tilde{P}}_{E_{IF}}=0$$

(37) $$\rho_{G}+d{\tilde{t}}_{rG\Phi }+m_{f}zpd_{w}+N_{G_{IF}}+\varepsilon_{\Phi G_{IF}}-\Phi_{G_{IF}}=0$$

(38) $$\rho_{E}+d{\tilde{t}}_{rG\Phi }+m_{f}zpd_{w}+N_{E_{IF}}+ISB_{C}+\varepsilon_{\Phi E_{IF}}+\Phi_{E_{IF}}=0$$

The carrier phase satellite hardware delays will be lumped to the ambiguity parameters as shown in Equations (39) and (40):

(39) $${\tilde{N}}_{G_{IF}}=\lambda_{G_{IF}}[f_{1}N_{1}-f_{2}N_{2}]+b_{\Phi }^{S}$$

(40) $${\tilde{N}}_{E_{IF}}=\lambda_{E_{IF}}[f_{E1}N_{1}-f_{E5a}N_{2}]+b_{E\Phi }^{S}$$

## 3. BSSD GPS/Galileo Models

### 3.1. Traditional BSSD GPS/Galileo PPP Model

As indicated earlier, two scenarios are considered when forming the BSSD linear combination, namely a tight and a loose combination. In the first scenario, either a GPS or a Galileo satellite is selected as a reference for both GPS and Galileo observables [6]. Taking a GPS satellite as a reference and using Equations (11)–(14), we obtain:

(41) $$\rho_{G,G}^{ij}+m_{f}^{ij}zpd_{w}+{\tilde{\varepsilon }}_{PG_{IF}}^{ij}-{\tilde{P}}_{G_{IF}}^{ij}=0$$

(42) $$\rho_{E,G}^{ik}+m_{f}^{ik}zpd_{w}+ISB+{\tilde{\varepsilon }}_{PE_{IF}}^{ik}-{\tilde{P}}_{EG_{IF}}^{ik}=0$$

(43) $$\rho_{G,G}^{ij}+m_{f}^{ij}zpd_{w}+{\tilde{N}}_{G_{IF}}^{ij}+{\tilde{\varepsilon }}_{\Phi G_{IF}}^{ij}-{\tilde{\Phi }}_{G_{IF}}^{ij}=0$$

(44) $$\rho_{E,G}^{ik}+m_{f}^{ik}zpd_{w}+ISB+{\tilde{N}}_{EG_{IF}}^{ik}+{\tilde{\varepsilon }}_{\Phi E_{IF}}^{ik}-{\tilde{\Phi }}_{EG_{IF}}^{ik}=0$$

where, the superscript *i* refers to the GPS reference satellite; the superscripts *j* and *k* refer the GPS and Galileo satellites respectively; ${\tilde{N}}_{G_{IF}}^{ij}$ and ${\tilde{N}}_{EG_{IF}}^{ik}$ are given by:

(45) $${\tilde{N}}_{G_{IF}}^{ij}=N_{G_{IF}}^{i}-N_{G_{IF}}^{j}+b_{G\Phi }^{ij}-b_{P}^{ij}$$

(46) $${\tilde{N}}_{EG_{IF}}^{ik}=N_{G_{IF}}^{i}-N_{E_{IF}}^{k}+b_{r_{E\Phi }}-b_{r_{\Phi }}+b_{E\Phi }^{K}-b_{\Phi }^{i}+b_{P}^{i}-b_{E}^{K}$$

As shown in Equation (45), the GPS ambiguity parameters include only the GPS satellite hardware delays. However, for Galileo system, the ambiguity parameters include both of the receiver and satellite hardware delays. Similarly, when a Galileo satellite is selected as a reference, using Equations (11)–(14) leads to:

(47) $$\rho_{G,E}^{lj}+m_{f}^{lj}zpd_{w}-ISB+{\tilde{\varepsilon }}_{PG_{IF}}^{lj}-{\tilde{P}}_{GE_{IF}}^{lj}=0$$

(48) $$\rho_{E,E}^{lk}+m_{f}^{lk}zpd_{w}+{\tilde{\varepsilon }}_{PE_{IF}}^{lk}-{\tilde{P}}_{E_{IF}}^{lk}=0$$

(49) $$\rho_{G,E}^{lj}+m_{f}^{lj}zpd_{w}-ISB+{\tilde{N}}_{GE_{IF}}^{lj}+{\tilde{\varepsilon }}_{\Phi G_{IF}}^{lj}-{\tilde{\Phi }}_{GE_{IF}}^{lj}=0$$

(50) $$\rho_{E,E}^{lk}+m_{f}^{lk}zpd_{w}+{\tilde{N}}_{E_{IF}}^{lk}+{\tilde{\varepsilon }}_{\Phi E_{IF}}^{lk}-{\tilde{\Phi }}_{E_{IF}}^{lk}=0$$

where, the superscript *l* refers to the Galileo reference satellite, ${\tilde{N}}_{GE_{IF}}^{lj}$ and ${\tilde{N}}_{E_{IF}}^{lk}$ are the BSSD non-integer ambiguity parameters lumped to the receiver and satellite hardware delays, which are given by:

(51) $${\tilde{N}}_{GE_{IF}}^{lj}=N_{E_{IF}}^{l}-N_{G_{IF}}^{j}+b_{r_{G\Phi }}-b_{r_{E\Phi }}+b_{G\Phi }^{j}-b_{E\Phi }^{l}+b_{E}^{l}-b_{P}^{j}$$

(52) $${\tilde{N}}_{E_{IF}}^{lk}=N_{E_{IF}}^{l}-N_{E_{IF}}^{k}+b_{E\Phi }^{lk}-b_{E}^{lk}$$

In the loose BSSD combination, two reference satellites are considered: a GPS reference satellite for the GPS observables and a Galileo satellite for the Galileo observables. Using Equations (11)–(14), we obtain:

(53) $$\rho_{G,G}^{ij}+m_{f}^{ij}zpd_{w}+{\tilde{\varepsilon }}_{PG_{IF}}^{ij}-{\tilde{P}}_{G_{IF}}^{ij}=0$$

(54) $$\rho_{E,E}^{lk}+m_{f}^{lk}zpd_{w}+{\tilde{\varepsilon }}_{PE_{IF}}^{lk}-{\tilde{P}}_{E_{IF}}^{lk}=0$$

(55) $$\rho_{G,G}^{ij}+m_{f}^{ij}zpd_{w}+{\tilde{N}}_{G_{IF}}^{ij}+{\tilde{\varepsilon }}_{\Phi G_{IF}}^{ij}-{\tilde{\Phi }}_{G_{IF}}^{ij}=0$$

(56) $$\rho_{E,E}^{lk}+m_{f}^{lk}zpd_{w}+{\tilde{N}}_{E_{IF}}^{lk}+{\tilde{\varepsilon }}_{\Phi E_{IF}}^{lk}-{\tilde{\Phi }}_{E_{IF}}^{lk}=0$$

where, ${\tilde{N}}_{G_{IF}}^{ij}$ and ${\tilde{N}}_{E_{IF}}^{lk}$ are the BSSD non-integer ambiguity parameters lumped to the receiver and satellite hardware delays as shown in Equations (57) and (58):

(57) $${\tilde{N}}_{G_{IF}}^{ij}=N_{G_{IF}}^{i}-N_{G_{IF}}^{j}+b_{G\Phi }^{ij}-b_{P}^{ij}$$

(58) $${\tilde{N}}_{E_{IF}}^{lk}=N_{E_{IF}}^{l}-N_{E_{IF}}^{k}+b_{E\Phi }^{lk}-b_{E}^{lk}$$

In this case, all receiver hardware delays are canceled out for both systems. The major advantage of the above per-constellation system is that both of the receiver clock error and the inter-system bias are cancelled out.

### 3.2. BSSD Decoupled Clock GPS/Galileo PPP Model

The BSSD decoupled clock model can be formed by using a GPS satellite as a reference. Using Equations (29)–(32), we obtain:

(59) $$\rho_{G,G}^{ij}+m_{f}^{ij}zpd_{w}+{\tilde{\varepsilon }}_{PG_{IF}}^{ij}-{\tilde{P}}_{G_{IF}}^{ij}=0$$

(60) $$\rho_{E,G}^{ik}+m_{f}^{ik}zpd_{w}+ISB_{P}+{\tilde{\varepsilon }}_{PE_{IF}}^{ik}-{\tilde{P}}_{EG_{IF}}^{ik}=0$$

(61) $$\rho_{G,G}^{ij}+m_{f}^{ij}zpd_{w}+{\tilde{N}}_{G_{IF}}^{ij}+{\tilde{\varepsilon }}_{\Phi G_{IF}}^{ij}-{\tilde{\Phi }}_{G_{IF}}^{ij}=0$$

(62) $$\rho_{E,G}^{ik}+m_{f}^{ik}zpd_{w}+ISB_{C}+{\tilde{N}}_{EG_{IF}}^{ik}+{\tilde{\varepsilon }}_{\Phi E_{IF}}^{ik}-{\tilde{\Phi }}_{EG_{IF}}^{ik}=0$$

where, ${\tilde{N}}_{G_{IF}}^{ij}$ and ${\tilde{N}}_{E_{IF}}^{ij}$ are given respectively by:

(63) $${\tilde{N}}_{G_{IF}}^{ij}=N_{G_{IF}}^{i}-N_{G_{IF}}^{j}$$

(64) $${\tilde{N}}_{EG_{IF}}^{ij}=N_{G_{IF}}^{i}-N_{E_{IF}}^{j}$$

When a Galileo satellite is selected as a reference, the BSSD equations take the form:

(65) $$\rho_{G,E}^{lj}+m_{f}^{lj}zpd_{w}-ISB_{P}+{\tilde{\varepsilon }}_{PG_{IF}}^{lj}-{\tilde{P}}_{GE_{IF}}^{lj}=0$$

(66) $$\rho_{E,E}^{lk}+m_{f}^{lk}zpd_{w}+{\tilde{\varepsilon }}_{PE_{IF}}^{lk}-{\tilde{P}}_{E_{IF}}^{lk}=0$$

(67) $$\rho_{G,E}^{lj}+m_{f}^{lj}zpd_{w}-ISB_{C}+{\tilde{N}}_{GE_{IF}}^{lj}+{\tilde{\varepsilon }}_{\Phi G_{IF}}^{lj}-{\tilde{\Phi }}_{GE_{IF}}^{lj}=0$$

(68) $$\rho_{E,E}^{lk}+m_{f}^{lk}zpd_{w}+{\tilde{N}}_{E_{IF}}^{lk}+{\tilde{\varepsilon }}_{\Phi E_{IF}}^{lk}-{\tilde{\Phi }}_{E_{IF}}^{lk}=0$$

where, ${\tilde{N}}_{GE_{IF}}^{lj}$ and ${\tilde{N}}_{E_{IF}}^{lk}$ are given by Equations (69) and (70), respectively:

(69) $${\tilde{N}}_{GE_{IF}}^{lj}=N_{E_{IF}}^{l}-N_{G_{IF}}^{j}$$

(70) $${\tilde{N}}_{E_{IF}}^{ik}=N_{E_{IF}}^{l}-N_{E_{IF}}^{k}$$

In the per-constellation BSSD model, two reference satellites are selected are references, a GPS and a Galileo. Using Equations (29)‒(32), we obtain:

(71) $$\rho_{G,G}^{ij}+m_{f}^{ij}zpd_{w}+{\tilde{\varepsilon }}_{PG_{IF}}^{ij}-{\tilde{P}}_{G_{IF}}^{ij}=0$$

(72) $$\rho_{E,E}^{lk}+m_{f}^{lk}zpd_{w}+{\tilde{\varepsilon }}_{PE_{IF}}^{lk}-{\tilde{P}}_{E_{IF}}^{lk}=0$$

(73) $$\rho_{G,G}^{ij}+m_{f}^{ij}zpd_{w}+{\tilde{N}}_{G_{IF}}^{ij}+{\tilde{\varepsilon }}_{\Phi G_{IF}}^{ij}-{\tilde{\Phi }}_{G_{IF}}^{ij}=0$$

(74) $$\rho_{E,E}^{lk}+m_{f}^{lk}zpd_{w}+{\tilde{N}}_{E_{IF}}^{lk}+{\tilde{\varepsilon }}_{\Phi E_{IF}}^{lk}-{\tilde{\Phi }}_{E_{IF}}^{lk}=0$$

As can be seen in Equations (75)‒(78), the *ISB* terms are cancelled. The differenced ambiguity parameters ${\tilde{N}}_{G_{IF}}^{ij}$ and ${\tilde{N}}_{E_{IF}}^{lk}$ can still be obtained from Equations (75) and (76), respectively.

(75) $${\tilde{N}}_{G_{IF}}^{ij}=N_{G_{IF}}^{i}-N_{G_{IF}}^{j}$$

(76) $${\tilde{N}}_{E_{IF}}^{ik}=N_{E_{IF}}^{l}-N_{E_{IF}}^{k}$$

### 3.3. BSSD Semi-Decoupled Clock GPS/Galileo PPP Model

The BSSD semi-decoupled clock model can be formed by using a GPS satellite as a reference. Using Equations (35)–(38), we obtain:

(77) $$\rho_{G,G}^{ij}+m_{f}^{ij}zpd_{w}+{\tilde{\varepsilon }}_{PG_{IF}}^{ij}-{\tilde{P}}_{G_{IF}}^{ij}=0$$

(78) $$\rho_{E,G}^{ik}+m_{f}^{ik}zpd_{w}+ISB_{P}+{\tilde{\varepsilon }}_{PE_{IF}}^{ik}-{\tilde{P}}_{EG_{IF}}^{ik}=0$$

(79) $$\rho_{G,G}^{ij}+m_{f}^{ij}zpd_{w}+{\tilde{N}}_{G_{IF}}^{ij}+{\tilde{\varepsilon }}_{\Phi G_{IF}}^{ij}-{\tilde{\Phi }}_{G_{IF}}^{ij}=0$$

(80) $$\rho_{E,G}^{ik}+m_{f}^{ik}zpd_{w}+ISB_{C}+{\tilde{N}}_{EG_{IF}}^{ik}+{\tilde{\varepsilon }}_{\Phi E_{IF}}^{ik}-{\tilde{\Phi }}_{EG_{IF}}^{ik}=0$$

where, ${\tilde{N}}_{G_{IF}}^{ij}$ and ${\tilde{N}}_{EG_{IF}}^{ik}$ are given respectively by:

(81) $${\tilde{N}}_{G_{IF}}^{ij}=N_{G_{IF}}^{i}-N_{G_{IF}}^{j}+b_{\Phi }^{i}-b_{\Phi }^{j}$$

(82) $${\tilde{N}}_{EG_{IF}}^{ij}=N_{E_{IF}}^{i}-N_{G_{IF}}^{j}+b_{\Phi }^{i}-b_{E\Phi }^{j}$$

When a Galileo satellite is selected as a reference, the BSSD equations take the form:

(83) $$\rho_{G,E}^{lj}+m_{f}^{lj}zpd_{w}-ISB_{P}+{\tilde{\varepsilon }}_{PG_{IF}}^{lj}-{\tilde{P}}_{GE_{IF}}^{lj}=0$$

(84) $$\rho_{E,E}^{lk}+m_{f}^{lk}zpd_{w}+{\tilde{\varepsilon }}_{PE_{IF}}^{lk}-{\tilde{P}}_{E_{IF}}^{lk}=0$$

(85) $$\rho_{G,E}^{lj}+m_{f}^{lj}zpd_{w}-ISB_{C}+{\tilde{N}}_{GE_{IF}}^{lj}+{\tilde{\varepsilon }}_{\Phi G_{IF}}^{lj}-{\tilde{\Phi }}_{GE_{IF}}^{lj}=0$$

(86) $$\rho_{E,E}^{lk}+m_{f}^{lk}zpd_{w}+{\tilde{N}}_{E_{IF}}^{lk}+{\tilde{\varepsilon }}_{\Phi E_{IF}}^{lk}-{\tilde{\Phi }}_{E_{IF}}^{lk}=0$$

where, ${\tilde{N}}_{GE_{IF}}^{lj}$ and ${\tilde{N}}_{E_{IF}}^{lk}$ are given by Equations (87) and (88), respectively:

(87) $${\tilde{N}}_{GE_{IF}}^{lj}=N_{E_{IF}}^{l}-N_{G_{IF}}^{j}+b_{E\Phi }^{l}-b_{\Phi }^{j}$$

(88) $${\tilde{N}}_{E_{IF}}^{lk}=N_{E_{IF}}^{l}-N_{E_{IF}}^{k}+b_{E\Phi }^{l}-b_{E\Phi }^{k}$$

In the per-constellation BSSD model, two reference satellites are selected are references, a GPS and a Galileo. Using Equations (35)–(38), we obtain:

(89) $$\rho_{G,G}^{ij}+m_{f}^{ij}zpd_{w}+{\tilde{\varepsilon }}_{PG_{IF}}^{ij}-{\tilde{P}}_{G_{IF}}^{ij}=0$$

(90) $$\rho_{E,E}^{lk}+m_{f}^{lk}zpd_{w}+{\tilde{\varepsilon }}_{PE_{IF}}^{lk}-{\tilde{P}}_{E_{IF}}^{lk}=0$$

(91) $$\rho_{G,G}^{ij}+m_{f}^{ij}zpd_{w}+{\tilde{N}}_{G_{IF}}^{ij}+{\tilde{\varepsilon }}_{\Phi G_{IF}}^{ij}-{\tilde{\Phi }}_{G_{IF}}^{ij}=0$$

(92) $$\rho_{E,E}^{lk}+m_{f}^{lk}zpd_{w}+{\tilde{N}}_{E_{IF}}^{lk}+{\tilde{\varepsilon }}_{\Phi E_{IF}}^{lk}-{\tilde{\Phi }}_{E_{IF}}^{lk}=0$$

As can be seen in Equations (89)–(92), the *ISB* terms are cancelled out. The differenced ambiguity parameters ${\tilde{N}}_{G_{IF}}^{ij}$ and ${\tilde{N}}_{E_{IF}}^{lk}$ can still be obtained through Equations (93) and (94), respectively.

(93) $${\tilde{N}}_{G_{IF}}^{ij}=N_{G_{IF}}^{i}-N_{G_{IF}}^{j}+b_{\Phi }^{i}-b_{\Phi }^{j}$$

(94) $${\tilde{N}}_{E_{IF}}^{lk}=N_{E_{IF}}^{l}-N_{E_{IF}}^{k}+b_{E\Phi }^{l}-b_{E\Phi }^{k}$$

## 4. Least Squares Estimation Technique

Under the assumption that the observations are uncorrelated and the errors are normally distributed with zero mean, the covariance matrix of the un-differenced observations takes the form of a diagonal matrix. The elements along the diagonal line represent the variances of the code and carrier phase measurements. Following the common practice, the ratio between the standard deviations of the code and the carrier-phase measurements is taken as 100. When forming BSSD, however, the differenced observations become mathematically correlated. This leads to a fully populated covariance matrix at a particular epoch.

The general linearized form for the above observation equations around the initial (approximate) vector ***u***^0^ and observables **𝑙** can be written in a compact form as:

(95) f(u,l)≈AΔu−w−r≈0

where ***u*** is the vector of unknown parameters; ***A*** is the design matrix, which includes the partial derivatives of the observation equations with respect to the unknown parameters ***u***; **Δ*u*** is the unknown vector of corrections to the approximate parameters ***u***^0^, *i.e*., ***u*** = ***u***^0^ + **Δ*u***; ***w*** is the misclosure vector and ***r*** is the vector of residuals. The sequential least-squares solution for the unknown parameters **Δ*u_i_*** at an epoch *i* can be obtained from [26]:

(96) $$\Delta u_{i}=\Delta u_{i-1}+M_{i-1}^{-1}A_{i}^{T}{\left(C_{l_{i}}+A_{i}M_{i-1}^{-1}A_{i}^{T}\right)}^{-1}[w_{i}-A_{i}\Delta u_{i-1}]$$

(97) $$M_{i}^{-1}=M_{i-1}^{-1}-M_{i-1}^{-1}A_{i}^{T}{\left(C_{l_{i}}+A_{i}M_{i-1}^{-1}A^{T}\right)}^{-1}A_{i}M_{i-1}^{-1}$$

(98) $$C_{\Delta u_{i}}=M_{i}^{-1}=M_{i-1}^{-1}-M_{i-1}^{-1}A_{i}^{T}{\left(C_{l_{i}}+A_{i}M_{i-1}^{-1}A^{T}\right)}^{-1}A_{i}M_{i-1}^{-1}$$

where **Δ*u***_*i−1*_ is the least-squares solution for the estimated parameters at epoch *i − 1*; ***M*** is the matrix of the normal equations; ***C****_l_* and ***C***_Δ*u*_ are the covariance matrices of the observations and unknown parameters, respectively. It should be pointed out that the usual batch least-squares adjustment should be used in the first epoch, *i.e*., for *i* = 1. The batch solution for the estimated parameters and the inverse of the normal equation matrix are given, respectively, by [26]:

(99) $$\Delta u_{1}={\left[C_{x^{0}}^{-1}+A_{1}^{T}C_{l_{1}}^{-1}A_{1}\right]}^{-1}A_{1}^{T}C_{l_{1}}^{-1}w_{1}$$

(100) $$M_{1}^{-1}={\left[C_{x^{0}}^{-1}+A_{1}^{T}C_{l_{1}}^{-1}A_{1}\right]}^{-1}$$

where ***C****_x_^0^* is the *a priori* covariance matrix for the approximate values of the unknown parameters.

In case of the traditional GPS/Galileo PPP model, the design matrix *A* and the vector of corrections to the unknown parameters Δ*x* take the following forms:

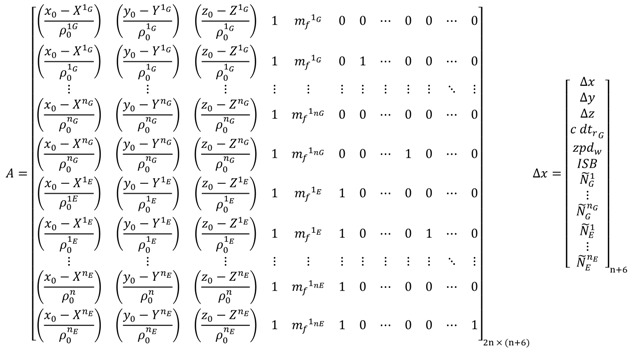
(101) where *n_G_* refers to the number of visible GPS satellites; *n_E_* refers to the number of visible Galileo satellites; *n* = *n_G_* + *n_E_* is the total number of the observed satellites for both GPS/Galileo systems; *x*_0_, *y*_0_ and *z*_0_ are the approximate receiver coordinates; $X^{j_{G}},\;Y^{j_{G}},\;Z^{j_{G}}=1,\;2,\;\ldots ,\;n_{G}$ are the known GPS satellite coordinates; $X^{k_{E}},\;Y^{k_{E}},\;Z^{k_{E}},\;k=1,\;2,\;\ldots ,\;n_{E}$ are the known Galileo satellite coordinates; *ρ*_0_ is the approximate receiver-satellite range. The unknown parameters in the above system are the corrections to the receiver coordinates, Δ*x*, Δ*y*, and Δ*z*, the wet component of the tropospheric zenith path delay *zpd_w_*, the inter-system bias *ISB*, and the non-integer ambiguity parameters $\tilde{N}$.

For the decoupled clock model, the design matrix *A* and the vector of corrections to the unknown parameters Δ*x* take the following forms: 
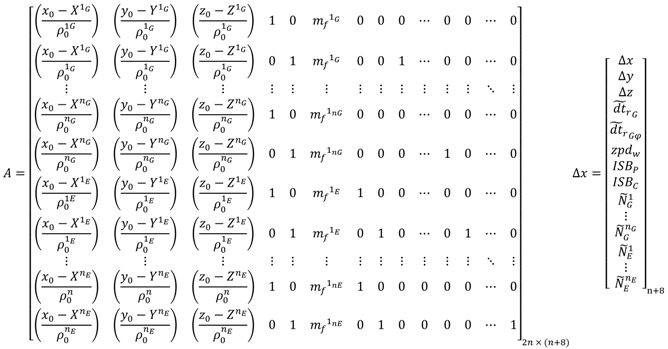
(102) where ${\tilde{dt}}_{r_{G}}$ and ${\tilde{dt}}_{r_{G\Phi }}$ are the pseudorange and carrier phase receiver clock errors, respectively; $ISB_{P}$ and $ISB_{C}$ are the pseudorange and carrier phase inter-system bias, respectively.

For the un-differenced semi-decoupled clock GPS/Galileo PPP model, the design matrix ***A*** and the vector of corrections to the unknown parameters Δ*x* take the same form as the decoupled clock GPS/Galileo model given in Equation (102). When a GPS satellite is selected as a reference to form the BSSD for GPS/Galileo observations, the design matrix ***A*** and the vector of corrections **Δ*u*** take the form: 
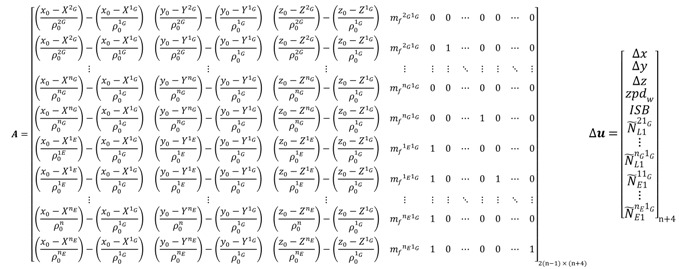
(103) where “*1_G_*” refers to the GPS reference satellite.

By analogy, the use of a Galileo satellite as a reference to form the BSSD for both of the GPS and Galileo observations leads to:

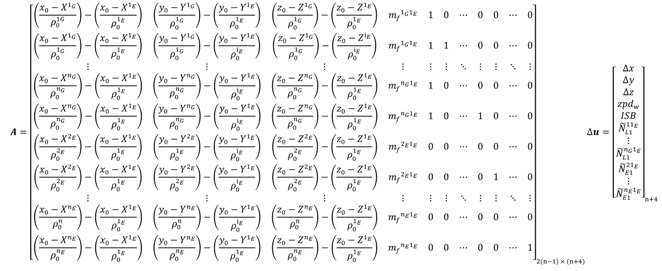
(104) where *1_E_* refers to the Galileo reference satellite.

When two reference satellites are selected to form the BSSD, *i.e*., per-constellation BSSD, the design matrix ***A*** and the vector of corrections **Δ*u*** take the form: 
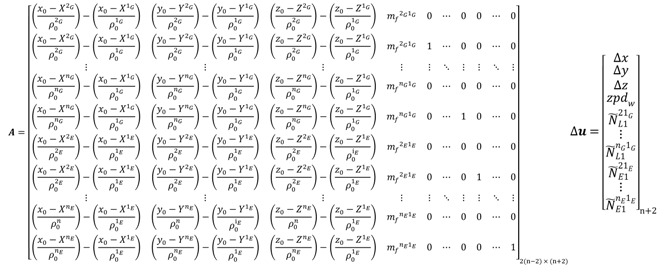
(105)

The major advantage of the above per-constellation (or loose combination) system is that the modified receiver clock error and the inter-system bias are cancelled out. Similarly, the design matrix ***A*** and the vector of corrections **Δ*u*** for the BSSD decoupled clock model, with a GPS satellite selected as a reference, take the form:

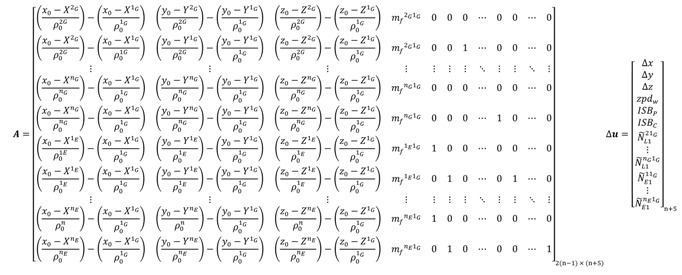
(106) where “*1_G_*” refers to the GPS reference satellite.

If, however, a Galileo satellite is selected as a reference, the design matrix ***A*** and the vector of corrections **Δ*u*** for the BSSD decoupled clock model take the form: 
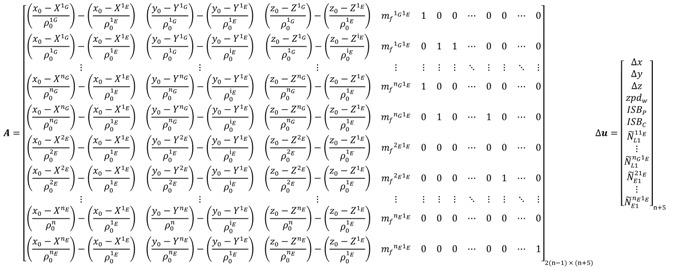
(107) where *1_E_* refers to the Galileo reference satellite. For the per-constellation BSSD decoupled clock model, the design matrix ***A*** and the vector of corrections **Δ*u*** will take the same form as those of the traditional BSSD GPS/Galileo PPP model. For the BSSD semi-decoupled clock GPS/Galileo PPP model, the design matrix ***A*** and the vector of corrections to the unknown parameters Δ*x* will be the same as those of the BSSD decoupled clock model.

## 5. Results and Discussion

**Figure 6:**
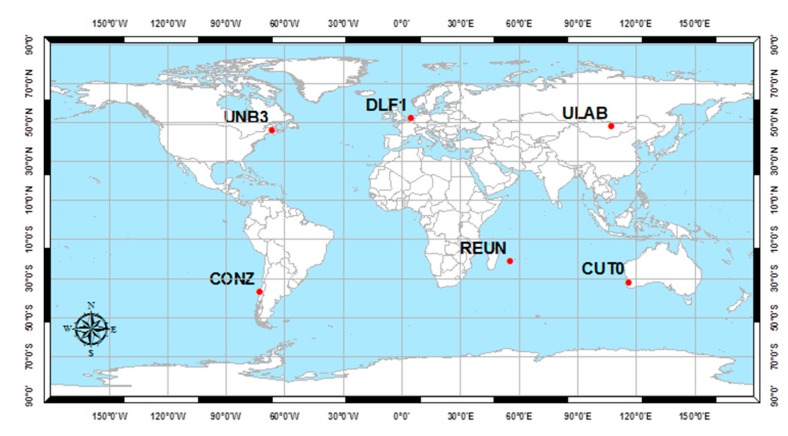
Analysis stations.

To verify the introduced GPS/Galileo PPP models, GPS/Galileo measurements at six well-distributed stations (Figure 6) were selected from the IGS tracking network [23]. Those stations are occupied by GNSS receivers, which are capable of simultaneously tracking the GPS/Galileo constellations. The positioning results for station DLF1 are presented below. Similar results are obtained from the other stations. However, a summary of the convergence times and precision are presented below for all stations. Natural Resources Canada (NRCan) GPSPace PPP software was modified to enable a GPS/Galileo PPP solution as described above. A solution is considered to be converged when the three-dimensional positioning standard deviation reaches 10 cm. The sampling interval for all data sets is 30 s of 5 April 2013, while the time span used in the analysis is one hour, which is selected to ensure that the four Galileo satellites are visible at each station.

**Figure 7:**
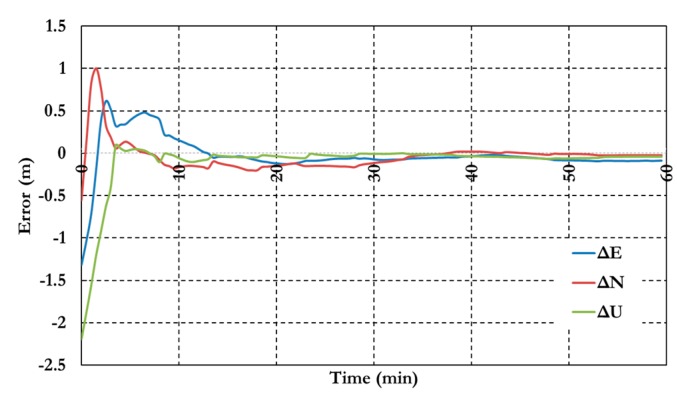
Postioining results of the traditional GPS/Galileo PPP model.

**Figure 8:**
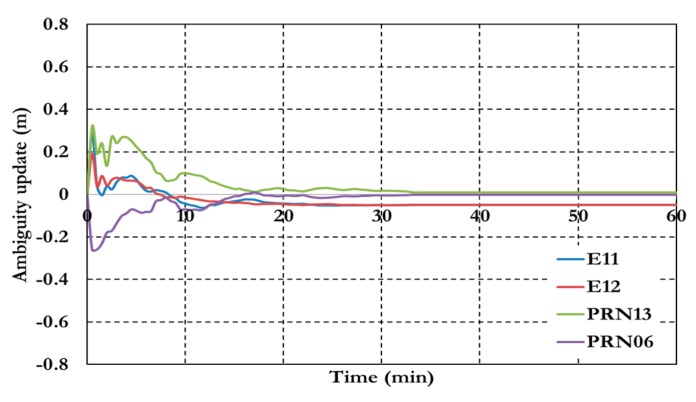
Ambiguity parameters the traditional GPS/Galileo PPP model.

Figure 7 and Figure 8 show the positioning results and the estimated ambiguity parameters of the traditional PPP model using GPS/Galileo observations.

**Figure 9:**
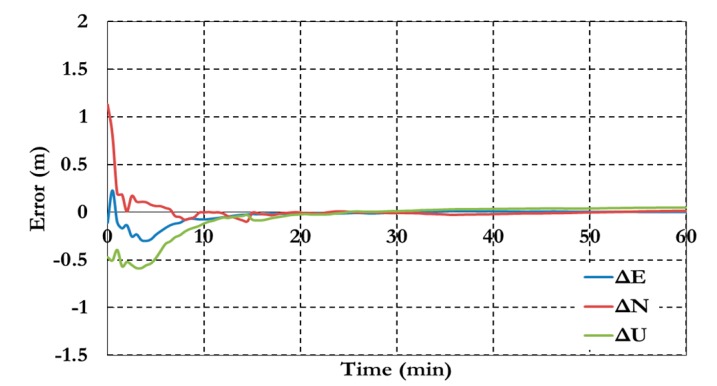
Positioning results of the GPS decoupled clock model.

**Figure 10:**
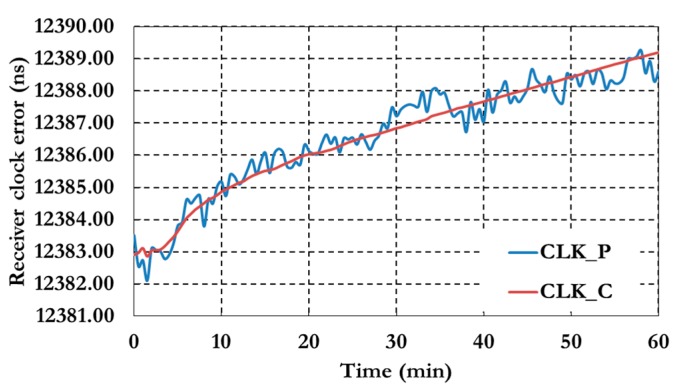
Receiver clock errors of the GPS decoupled clock model.

**Figure 11:**
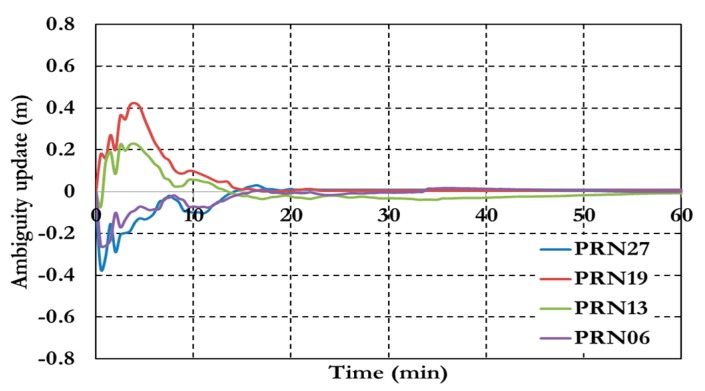
Ambiguity parameters of the GPS decoupled clock model.

As shown in Figure 6, the positioning results of the combined GPS/Galileo traditional PPP model have a convergence time of 15 min to reach decimeter-level precision. The ambiguity parameters results in Figure 8 shows that the un-calibrated hardware delayed that lumped to the ambiguity parameter affects the ambiguity parameters convergence. Figure 9, Figure 10 and Figure 11 show the results of the GPS decoupled clock model.

Figure 9 shows the positioning results of the decoupled clock model. The results show a decimeter level of precision with about 15 min. Generally the precision of the decoupled clock model positioning results are about 25% more than the traditional PPP model. Figure 10 shows the receiver clock errors for both pseudorange (CLK_P) and carrier phase (CLK_C) observation.

Figure 11 shows the results of the ambiguity parameters of the GPS decoupled clock model.

**Figure 12:**
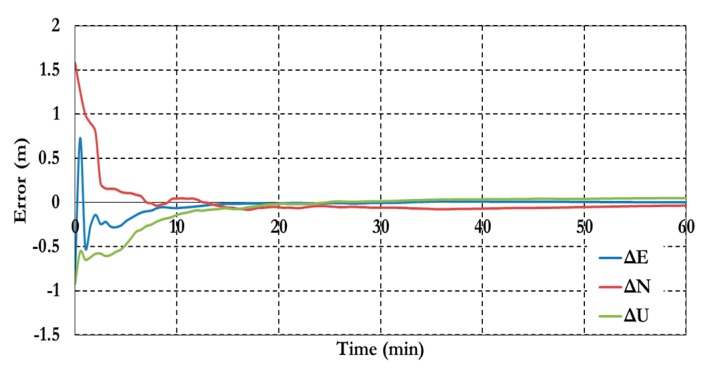
Positioning results of the semi-decoupled clock PPP model.

**Figure 13:**
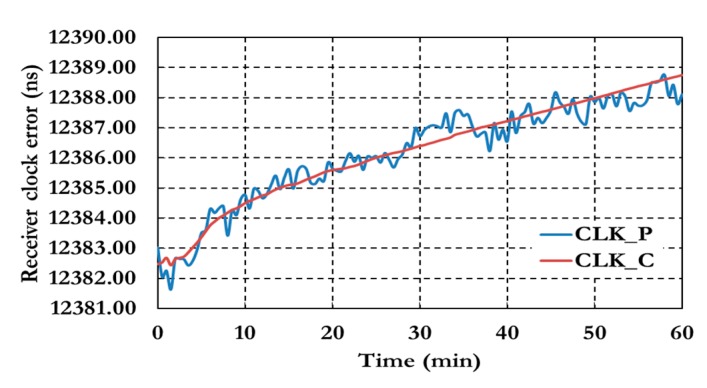
Receiver clock errors of the semi-decoupled clock GPS/Galileo PPP model.

**Figure 14:**
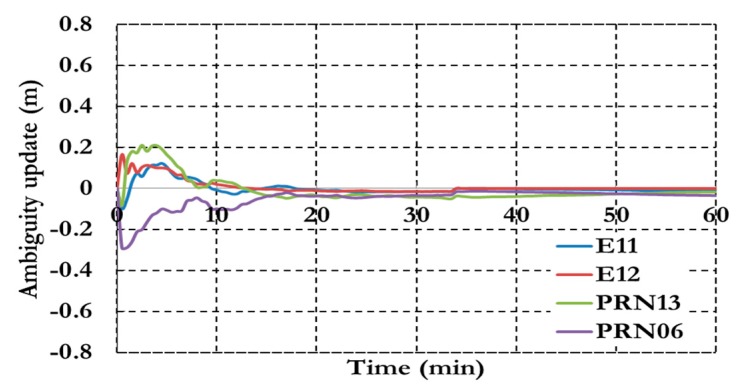
Ambiguity parameters of the semi-decoupled clock GPS/Galileo PPP model.

**Figure 15:**
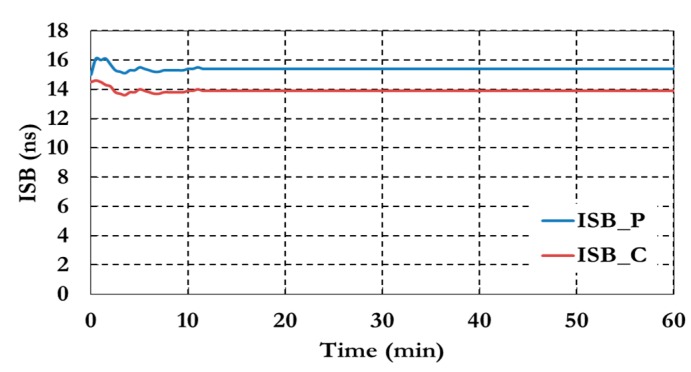
Inter-system bias of the semi-decoupled clock GPS/Galileo PPP model.

**Figure 16:**
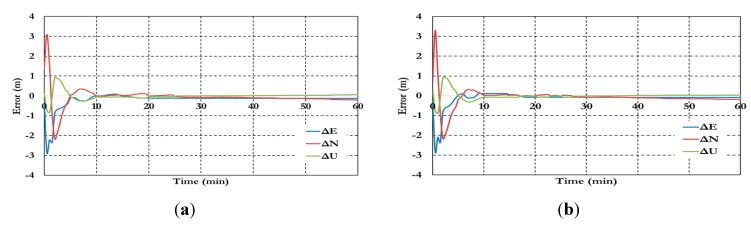
Positioning results of the BSSD PPP tight combination model using (**a**) GPS reference satellite; and (**b**) Galileo reference satellite

Figure 12, Figure 13, Figure 14 and Figure 15 show the results of the semi-decoupled clock GPS/Galileo PPP model. The positioning results in Figure 16 show that the semi-decoupled clock GPS/Galileo PPP model has a decimeter level of precision with about 15 min. In addition, the positioning precision of the semi-decoupled clock GPS/Galileo PPP model are improved by about 25% comparing to the traditional GPS/Galileo PPP model.

Figure 13 shows the receiver clock errors for both pseudorange (CLK_P) and carrier phase (CLK_C) observation.

Figure 14 shows the ambiguity parameters results of the semi-decoupled clock GPS/Galileo PPP model.

As shown in Figure 14 the ambiguity parameters results show a similar convergence time to the positioning results. Figure 15 shows the results of the inter-system bias parameters for both pseudorange (ISB_P) and carrier phase (ISB_C) observations.

**Figure 17:**
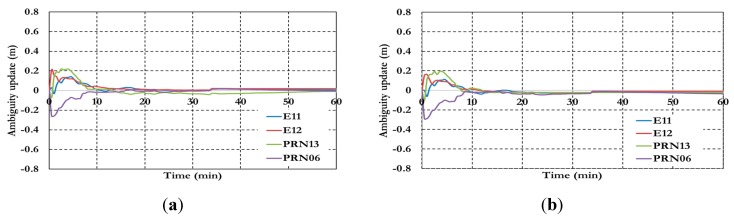
Ambiguity parameters the BSSD PPP tight combination model using (**a**) GPS reference satellite; and (**b**) Galileo reference satellite.

Figure 16 and Figure 17 show the BSSD PPP tight combination model positioning results and the estimated ambiguity parameters.

As shown in Figure 16, the positioning results of the BSSD tight combination model have convergence time of 10 min and decimeter level of precision.

**Figure 18:**
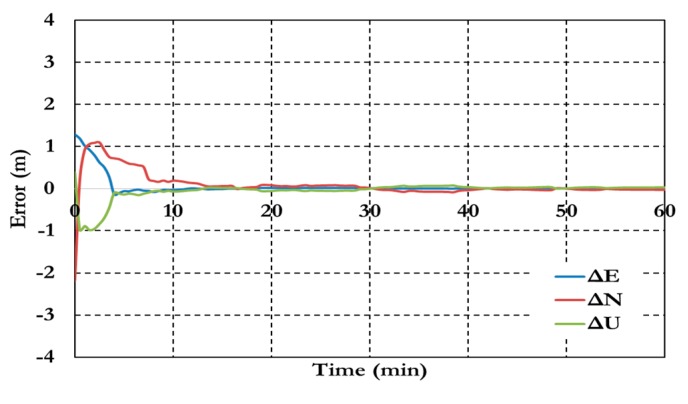
Positioning results of the BSSD PPP loose combination model.

**Figure 19:**
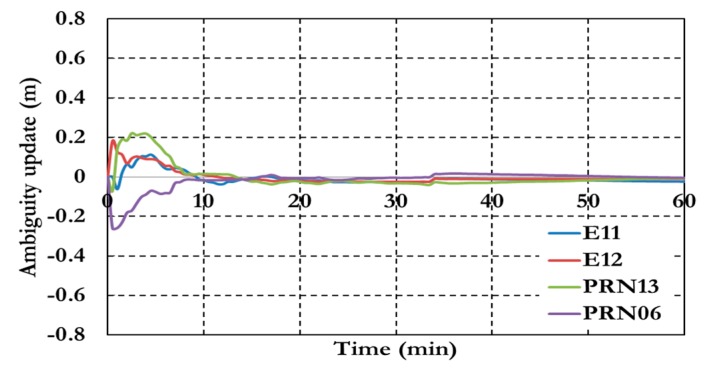
Ambiguity parameters the BSSD PPP loose combination model.

As shown in Figure 17, the results convergence of the ambiguity parameters are affected by the lumped DCB. Figure 18 and Figure 19 show the results of the BSSD PPP loose combination model for both positioning results and ambiguity parameters.

**Figure 20:**
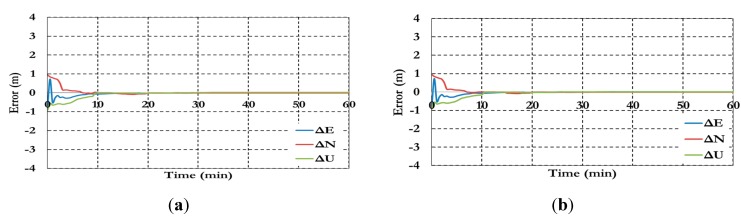
Positioning results for BSSD semi-decoupled GPS/Galileo PPP model. (**a**) GPS reference satellite; and (**b**) Galileo reference satellite.

**Figure 21:**
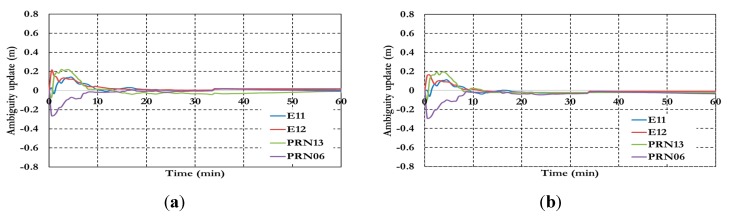
Ambiguity parameters the semi-decoupled GPS/Galileo PPP model (**a**) GPS reference satellite; and (**b**) Galileo reference satellite.

As shown in Figure 18, the positioning results show decimeter-level precision with about 11 min convergence time. Figure 19 shows that the ambiguity parameters of the BSSD loose combination model are affected by the lumped DCB. Figure 20 and Figure 21 show the positioning results and the estimated ambiguity parameters for the BSSD semi-decoupled GPS/Galileo PPP model, when a tight combination is used. As can be seen, the PPP solution convergences to a decimeter-level precision after about 10 min.

**Figure 22:**
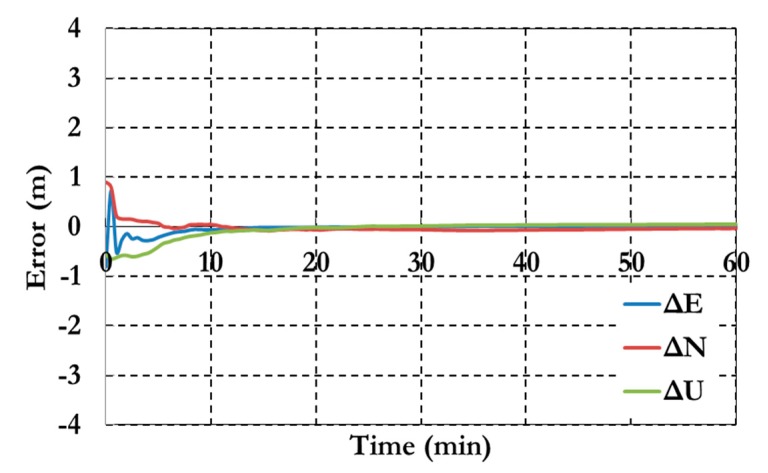
Positioning results of the semi-decoupled per-constellation GPS/Galileo BSSD PPP model.

**Figure 23:**
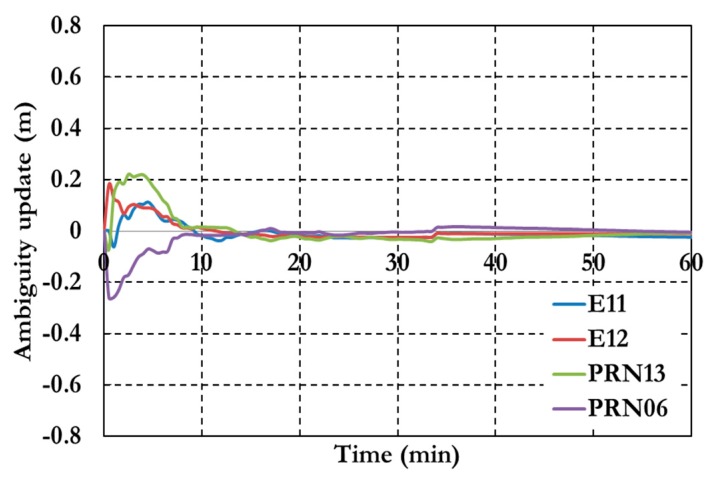
Ambiguity parameters the semi-decoupled per-constellation GPS/Galileo BSSD PPP model.

Figure 22 and Figure 23 show the results of the semi-decoupled per-constellation GPS/Galileo BSSD PPP model for both of the positioning and ambiguity parameters. As can be seen, the positioning results show decimeter-level precision with about 11 min convergence time.

**Figure 24:**
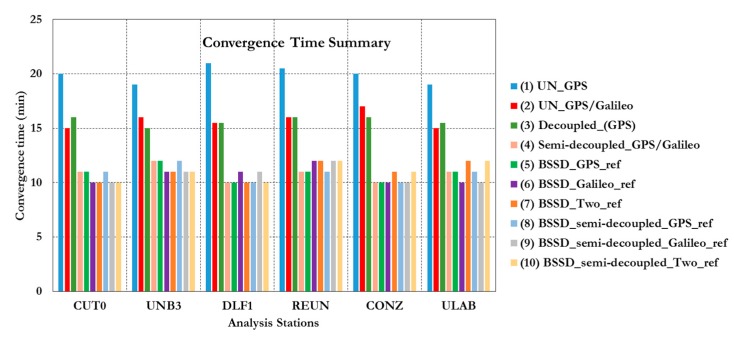
Summary of convergence times of all stations and analysis cases. (**1**) Un-differenced GPS model; (**2**) Un-differenced GPS/Galileo model; (**3**) Decoupled clock model using GPS observations only; (**4**) semi-decoupled clock GPS/Galileo PPP model; (**5**) BSSD model with a GPS satellite as a reference; (**6**) BSSD model with a Galileo satellite as a reference; (**7**) BSSD model with both a GPS and a Galileo satellite as reference satellites; (**8**) BSSD semi-decoupled clock GPS/Galileo model with a GPS satellite as a reference; (**9**) BSSD semi-decoupled clock GPS/Galileo model with a Galileo satellite as a reference; (**10**) BSSD semi-decoupled clock GPS/Galileo model with both a GPS and a Galileo satellite as reference satellites.

As shown in Figure 23, the ambiguity parameters results for both GPS and Galileo satellites are affected by the lumped DCB. Figure 24 summarizes the convergence times for all analysis cases, which confirm the PPP solution consistency at all stations.

**Figure 25:**
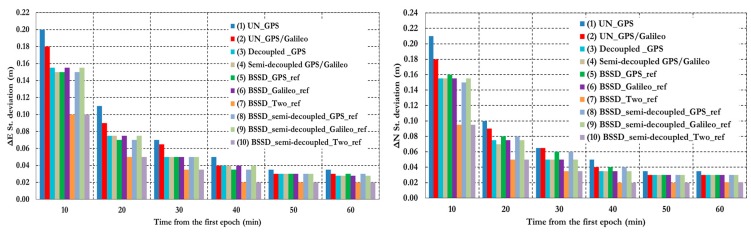

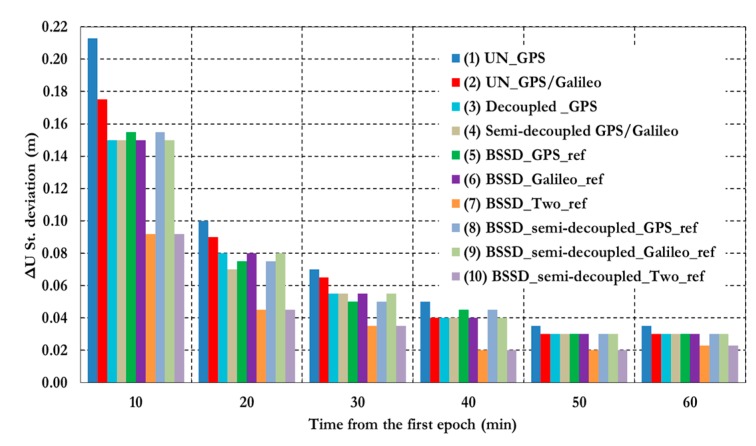
Summary of positioning standard deviations in East, North, and Up directions of all stations and analysis cases. (**1**) Un-differenced GPS model; (**2**) Un-differenced GPS/Galileo model; (**3**) Decoupled clock model using GPS observations only; (**4**) semi-decoupled clock GPS/Galileo PPP model; (**5**) BSSD model with a GPS satellite as a reference; (**6**) BSSD model with a Galileo satellite as a reference; (**7**) BSSD model with both a GPS and a Galileo satellite as reference satellites; (**8**) BSSD semi-decoupled clock GPS/Galileo model with a GPS satellite as a reference; (**9**) BSSD semi-decoupled clock GPS/Galileo model with a Galileo satellite as a reference; (**10**) BSSD semi-decoupled clock GPS/Galileo model with both a GPS and a Galileo satellite as reference satellites.

To further assess the performance of the various PPP models, the solution output is sampled every 10 min and the standard deviation of the computed station coordinates is calculated for each sample. Figure 25 shows the position standard deviations in the East, North, and Up directions, respectively. Examining the standard deviations after 10 min, it can be seen that the semi-decoupled clock GPS/Galileo PPP model improves the precision of the estimated parameters by about 25% in comparison with the un-differenced GPS-only model. As the number of epochs, and consequently the number of measurements, increases the performance of the various models tends to be comparable. An exception, however, is the loose combination model, which is found superior to all other PPP models.

## 6. Conclusions

This paper examined the performance of several PPP models, including the traditional un-differenced model, the decoupled clock model, the semi-decoupled clock model, and BSSD model. It has been shown that the traditional un-differenced GPS/Galileo PPP model, the GPS decoupled clock model, and the semi-decoupled clock GPS/Galileo PPP model improve the convergence time by about 25% in comparison with the traditional un-differenced GPS-only model. In addition, the semi-decoupled GPS/Galileo PPP model improves the solution precision by about 25% compared to the traditional un-differenced GPS/Galileo PPP model. Moreover, the BSSD GPS/Galileo PPP model improves the solution convergence time by about 50%, in comparison with the un-differenced GPS PPP model, regardless of the type of BSSD combination used. As well, the BSSD GPS/Galileo model improves the precision of the estimated parameters by about 50% and 25% when the loose and the tight combinations are used, respectively, in comparison with the traditional un-differenced GPS-only model. Comparable results are obtained through the tight combination when either a GPS or a Galileo satellite is selected as a reference.
